# Lack of resistance development in *Bemisia tabaci* to *Isaria fumosorosea* after multiple generations of selection

**DOI:** 10.1038/srep42727

**Published:** 2017-02-23

**Authors:** Tianni Gao, Zhaolei Wang, Yü Huang, Nemat O. Keyhani, Zhen Huang

**Affiliations:** 1 Department of Entomology, College of Agriculture, South China Agricultural University, Guangzhou, 510642, China; 2 Department of Microbiology and Cell Science, Institute of Food and Agricultural Sciences, University of Florida, Bldg. 981, Museum Rd., Gainesville, FL32611, USA

## Abstract

The emergence of insecticide resistant insect pests is of significant concern worldwide. The whitefly, *Bemisia tabaci*, is an important agricultural pest and has shown incredible resilience developing resistance to a number of chemical pesticides. Entomopathogenic fungi such as *Isaria fumosorosea* offer an attractive alternative to chemical pesticides for insect control, and this fungus has been shown to be an effective pathogen of *B. tabaci*. Little is known concerning the potential for the development of resistance to *I. fumosorosea* by *B. tabaci*. Five generations of successive survivors of *B. tabaci* infected by *I. fumosorosea* were assayed with *I. fumosorosea*. No significant differences in susceptibility to *I. fumosorosea*, number of ovarioles, or ovipostioning were seen between any of the generations tested. Effects of *I. fumosorosea* and cell-free ethyl acetate fractions derived from the fungus on the *B. tabaci* fat body, ovary, and vitellogenin were also investigated. These data revealed significant deformation and degradation of ovary tissues and associated vitellogenin by the fungal mycelium as well as by cell-free ethyl acetate fungal extracts. These data indicate the lack of the emergence of resistance to *I. fumosorosea* under the conditions tested and demonstrate invasion of the insect reproductive tissues during fungal infection.

The sweet potato whitefly, *Bemisia tabaci* (Gennadius) (Homoptera: Aleyrodidae), is one of the most destructive insect pests worldwide negatively affecting ornamental, vegetable, grain legume, and cotton production^1^,^2^,^3^. Damage to the host plant is caused by insect feeding on the phloem sap, with significant secondary effects resulting in infection of the plant by sooty molds that grow on the honeydew excreted by the insect as it feeds. *B. tabaci* can also act as a vector for the transmission of >600 different plant pathogenic viruses, infection by which can result in severe reductions in the photosynthetic ability of host plant, and hence stunt growth, decreasing crop yield and quality^4^,^5^,^6^,^7^. Chemical pesticides are the most commonly used method for suppressing *B. tabaci* populations^8^, however, the insect has been shown to rapidly develop insecticide resistance. This, coupled to the increasing environmental awareness of the hazards of chemical insecticides^9^,^10^,^11^, has promoted the development of alternative approaches. These include the use of biological control agents such as entomopathogenic fungi, that are more environmentally friendly and compatible with organic farming practices^12^,^13^,^14^,^15^. The use of fungal biological control agents has several advantages including: the low likelihood of resistance development, decreased non-target effects, and decreased human health and environmental impacts^16^,^17^,^18^,^19^. More than 20 species of entomopathogenic fungi are known to infect whiteflies^20^,^21^, including isolates of *Beauveria bassiana*^22^,^23^ and *Isaria fumosorosea*^24^,^25^,^26^.

*Isaria fumosorosea* (previously, *Paecilomyces fumosoroseus*^27^ is an important natural enemy of whiteflies including *B. tabaci*^13^,^24^,^25^,^28^. A number of studies have focused on the potential of *I. fumosorosea* to control adult and/or various larval stages of *P. xylostella, Trialeurodes vaporariorum* (Westwood), *Diaphorina citri*, and *B. tabaci*^12^,^24^,^29^,^30^,^31^,^32^,^33^. Although far more is known concerning the biochemical and molecular aspects of infection in the related *Beauveria* and *Metarhizium* species^34^,^35^, several studies have begun to examine these processes in *Isaria* sp^28^,^36^,^37^ including the isolation and characterization of several bioactive secondary metabolites^38^,^39^.

Aside from outright death of the host, sub-lethal effects of entomogenous fungi on insect fecundity and longevity has been explored^24^,^40^,^41^. Such effects can have important consequences for host population dynamics^42^,^43^, e.g. *I. fumosorosea* infection of *B. tabaci* and *P. xylostella* results in fewer eggs laid prior to death than control females of the same age^24^. An important consideration is the development of insect resistance to the agents employed for their control. Adaptation for resistance to chemical pesticides in a range of insect species, including *B. tabaci* is well documented^11^,^44^. Indeed, pesticide resistant *B. tabaci* can occur as rapidly as within few years, e.g. high resistance to pyriproxyfen (>1000-fold increase) has been reported within three years of use of the chemical pesticide^11^. Although some insects appear to be intrinsically more resistant to entomopathogenic fungi than others, e.g. amongst beetles, the red flour beetle *Tribolium castaneum* displays a relatively high level of resistance to *B. bassiana* in part due to the secretion of cuticular quinones^45^, there are no reports of the development of resistance to fungi used in biological control efforts in the field. This is likely due to the observation that infection of host is multi-modal, involving a combination of enzymes targeting degradation of the host, production of toxins and other secondary metabolites, and diverse mechanisms for overcoming the immune defenses of the host^35^,^46^,^47^. A report in which larvae of the greater waxmoth, *Galleria mellonella*, was under constant selective pressure from *B. bassiana* infection for 25 generations resulted in only a minor decrease in susceptibility (<10%), although changes in cuticular, humoral, and cellular defenses were noted between the 1^st^ and 25^th^ generations^48^. However, many insects that have been reported to have developed resistance to chemical pesticides continue to be susceptible to infection by entomopathogenic fungi, and in some cases have been reported to be more vulnerable than their wild type parents^49^,^50^,^51^,^52^,^53^.

Here, we investigated the potential of *B. tabaci* to develop resistance to *I. fumosorosea* under constant selective pressure from the fungus. In addition, insect fecundity and ovary development as well as reproductive tissue invasion by the fungus was monitored during infection. These data suggest that use of entomopathogenic fungi poses a low risk of resistance development and confirms their utility in pest control as part of Integrated Pest Management (IPM) practices.

## Results

### Susceptibility of *B. tacabi* selected under *I. fumosorosea* pressure to *I. fumosorosea*

**Figure 1:**
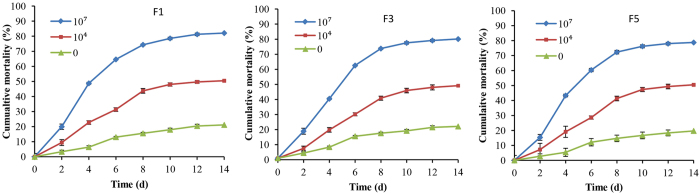
The cumulative mortality of *B. tabaci* nymph against *I. fumosorosea*. 0, 4, 7 mean the concentration of fungal conidia with 0, 1 × 10^4^, 1 × 10^7^ conidia/ml, respectively. F1, F3 and F5 mean the No. 1, 3 and 5 generation of *B. tabaci* nymph. Experiments were performed in triplicate, error bars = ±SE.

*B. tabaci* was selected under *I. fumosorosea* pressure for five generations, labeled F1 to F5, via iterative exposure of surviving insects to the fungal agent as detailed in the Methods section. The susceptibility of the F1, F3, and F5 generations to various concentrations of *I. fumosorosea* conidial suspensions was examined as described in the Methods section (Fig. 1). No significant differences were seen in the time course or mean cumulative mortalities of the different *B. tabaci* generations to either a low (10^4^ conidia/ml) or high dose (10^7^ conidia/ml) of the fungal agent (Table 1). The mean lethal time to kill (LT_50_) values for the high/low dose was 5.5 ± 0.5 d/28.1 ± 8.0 d, 6.3 ± 0.5 d/29.7 ± 7.8 d, and 6.4 ± 0.5 d/26.9 ± 6.8 d, for the F1, F3, and F5 *B. tabaci* generations, respectively, and were not significantly different.

### Effect of *I. fumosorosea* on *B. tabaci* ovipositioning

**Figure 2:**
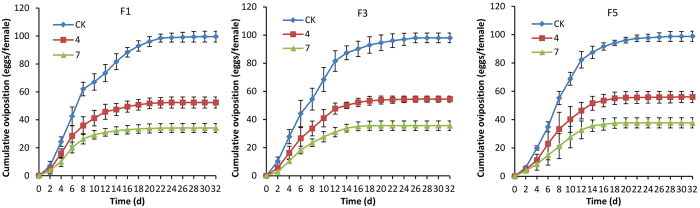
The cumulative oviposition of *B. tabaci* adult emergence from *I. fumosorosea*-selected *B. tabaci* nymph. CK, 4, 7 mean the concentration of fungal conidia with 0, 1 × 10^4^, 1 × 10^7^ conidia/ml, respectively. F1, F3 and F5 mean the No. 1, 3 and 5 generation of *B. tabaci* adult. Experiments were performed in triplicate, error bars = ±SE.

The effect of low (10^4^ conidia/ml) and high (10^7^ conidia/ml) doses of *I. fumosorosea* on the number of eggs laid by the F1, F3, and F5 generations of *I. fumosorosea*-selected *B. tabaci* generations was examined as detailed in the Methods section. A time course of the cumulative number of eggs laid by untreated, and low and high dose treared *B. tabaci* was performed (Fig. 2). Control (untreated) *B. tabaci* adult laid approximately 100 ± 4 eggs/female/30 d, with no significant differences seen between the F1, F3, and F5 generations. Infection by *I. fumorososea* even at the low dose (10^4^ conidia/ml) resulted in a sharp decrease (~50%) in the total number of eggs laid to ~55 ± 4 eggs/female/30 d when assayed against the F1, F3, and F5 *B. tabaci* generations, with equal reductions, i.e. no significant differences seen between the different generations (Table 2). Infections using the higher dose (10^7^ conidia/ml) resulted in a greater overall reduction in the number of eggs laid to ~35 ± 3 eggs/female/30 d, and similar to what was observed at the low dose, the reduction was similar across all of the *B. tabaci* selected generations, with no significant differences observed.

### Effect of *I. fumosorosea* on *B. tabaci* fat body, ovaries, and associated vitellogenin

**Figure 3:**
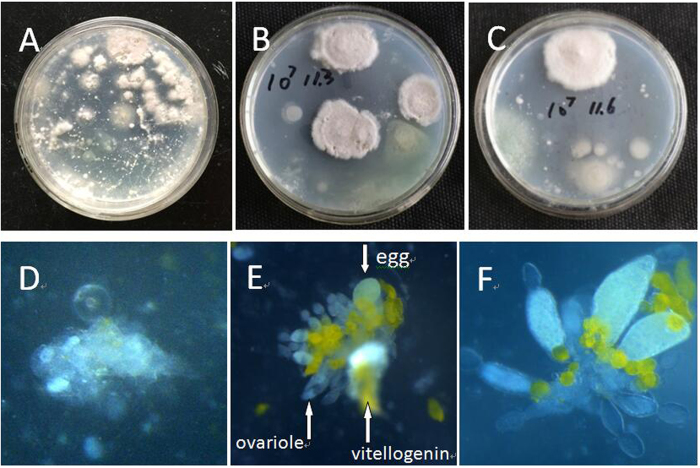
Fungal colony and ovary morphology of *B. tabaci* adult from different fungi treatment. (**A**) representative images of fungal colonies derived from dissected ovaries of *B. tabaci* adult at 2 d age, with adult emergence from *I. fumosorosea*-selected *B. tabaci* nymph. Dissected ovary were cultured on PDA medium; (**B**) colony from dissected ovaries of *B. tabaci* adult at 5 d age, and (**C**) colony from dissected ovaries of *B. tabaci* adult at 8 d age; (**D**) Representative image of deformity ovary in infected insects; (**E**,**F**) images of normal *B. tabaci* (untreated) ovaries (ovariole, egg and vitellogenin are marked with arrows).

In order to examine whether infection of *B. tabaci* by *I. fumosorosea* reached the ovaries during the normal progress of mycosis *in vivo*, the ovaries of *B. tabaci* newly emerged adults from topically nymph infected by *I. fumosorosea* (1 × 10^4^ and 1 × 10^7^ conidia/ml) were dissected and cultured on PDA. Fungal colonies could readily and continuously be isolate from dissected ovaries of adult at different ages cultured on PDA (Fig. 3A,B and C, images for 1 × 10^7^ conidia/ml infection shown). Microscopic observations revealed distortion and deformation of infected ovaries that was not seen in control *B. tabaci* ovaries (Fig. 3E and F). Degradation and almost complete loss of vitellogenin was also noted (Fig. 3D). Quantification of the number of ovarioles produced in the F1, F3, and F5 fungal-selected *B. tabaci* generations after infection with low (10^4^ conidia/ml) and high doses (10^7^ conidia/ml) of *I. fumosorosea* revealed (1) no significant effects with respect to infection, i.e. infection by either low or high doses of *I. fumosorosea* did not change the number of ovarioles seen as compared to control, and (2) no significant differences between the F1, F3, and F5 *B. tabaci* generations, the concentration of fungal inoculate used, or untreated controls (Table 3).

**Figure 4:**
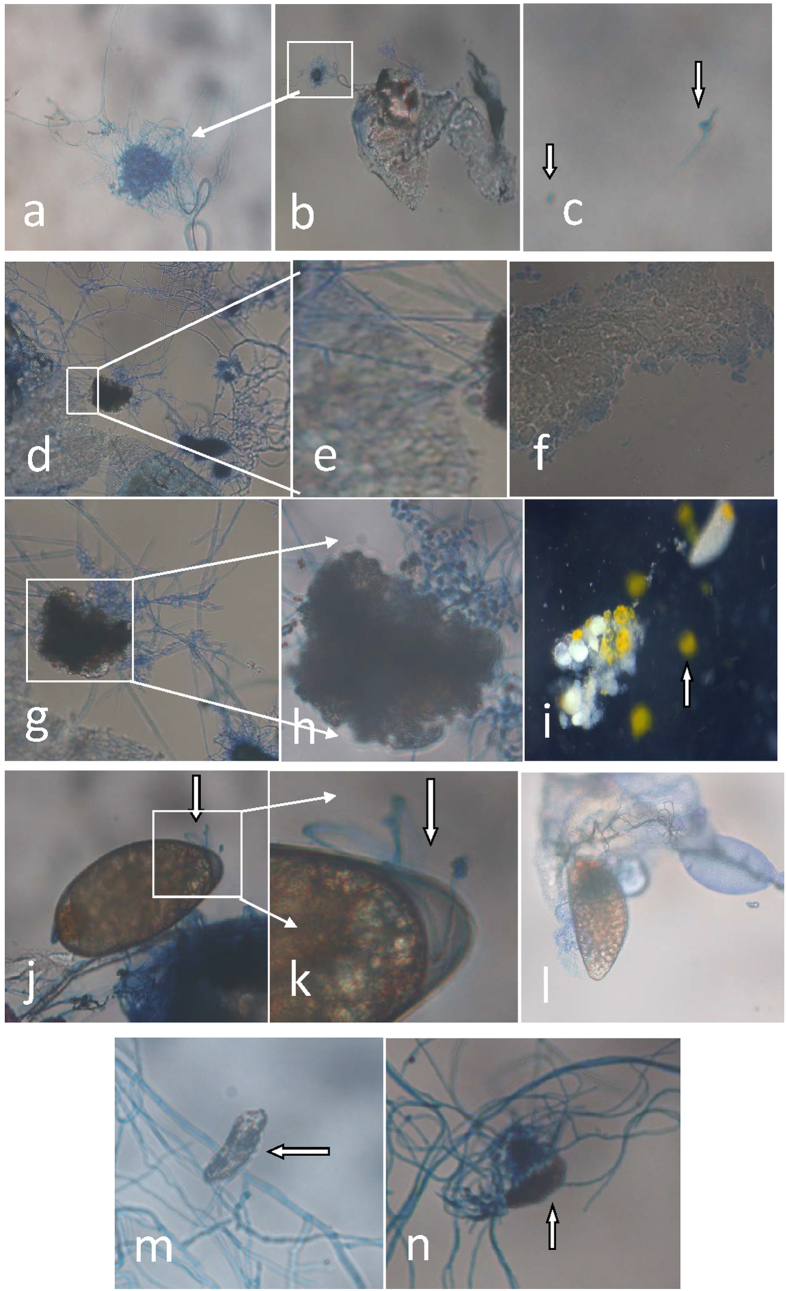
Representative images of *I. fumosorosea* growth on dissected *B. tabaci* fat body, egg and associated vitellogenin. (**a**,**b**) *I. fumosorosea* growth on dissected *B. tabaci* ovaries (12 h post-incubation), (**c**) *I. fumosorosea* growth in buffer solution alone (12 h post-incubation), (**d**,**e**) *I. fumosorosea* growth on dissected *B. tabaci* fat bodies (42 h post-incubation), (**f**) untreated *B. tabaci* fat body (42 h); (**g**,**h**) effect of *I. fumosorosea* on associated vitellogenin (42 h post-incubation), (**i**) control egg/vitellogenin samples (vitellogenin marked with arrow), (**j**,**k**) *I. fumosorosea* growth on dissected *B. tabaci* eggs (42 h post-incubation), (**m**,**n**) deformation of eggs due to *I. fumosorosea* infection (fungal mycelia marked with arrow, 42 h post-incubation).

In order to further examine the effect of fungal growth on the host reproductive system, dissected adult reproductive tissues were treated with fungal conidial suspensions. Dissected fat bodies and ovaries from healthy *B. tabaci* adults could readily support growth of *I. fumosorosea in vitro*. In the presence of dissected *B. tabaci* ovaries, *I. fumorosea* rapidly grew forming extended hyphae and emerging mycelia within 12 h of co-incubation (Fig. 4a,b). In contrast, little to no growth was seen in fungi kept in the buffer solution alone (Fig. 4c), and no microbial growth was evident in untreated dissected ovaries (Fig. 4i,l). Similarly, dissected fat bodies provided a good growth substrate for the fungus (Fig. 4d,e). Degradation of vitellogenin was also evident (Fig. 4g,h) in *in vitro* incubations of dissected tissues incubated with *I. fumosorosea*, as well as infection of dissected eggs (Fig. 4j,k,m,n).

### Effect of *I. fumosorosea* cell-free EthOAc extracts on dissected *B. tabaci* fat body, ovary, and associated vitellogenin

**Figure 5:**
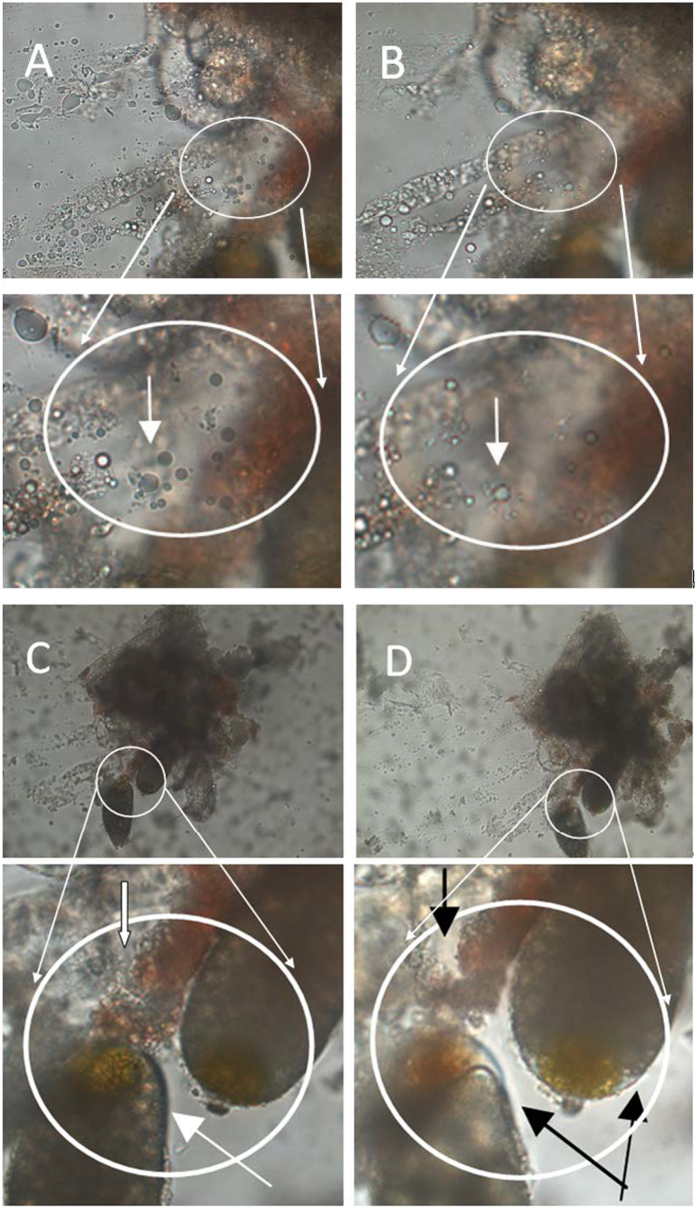
Effect of EtOAc extract on insect fat droplet, egg, and associated vitellogenin. (**A**) normal fat droplet, (**B**) fat droplet incubated in the presence of *I. fumosorosea* cell-free culture supernatant EtOAc extract (24 h post-incubation) showing changes in the overall morphology and number of droplets present (marked with arrows). Lower panels show enlargement of select regions. (**C**) Normal *B. tabaci* fat droplets, egg and associated vitellogenin, (**D**) *B. tabaci* fat droplet, egg and associated vitellogenin treated with *I. fumosorosea* EtOAc extract (24 h post-treatment) showing shrinkage/damage of the egg and reduction in fat droplets present (arrows). Representative images are shown.

We have previously shown that *I. fumosorosea* EthOAc extracts can be toxic to *B. tabaci*^54^. In order to examine whether these extracts can directly affect *B. tabaci* immune and reproductive tissues, healthy dissected *B. tabaci* fat bodies and ovaries were incubated in the presence of the *I. fumosorosea* cell-free EthOAc extracts and examined visually over time. Degradation of dissected *B. tabaci* fat bodies was evident as early as 6 h post treatment with clear disruption of the tissue seen by 24 h (Fig. 5A,B), whereas control untreated dissected fat bodies remained intact. Similarly, eggs treated with the cell-free *I. fumosorosea* extracts were significantly damaged and loss of vitellogenin was evident at 24 h post treatment, whereas control eggs were unaffected (Fig. 5C,D).

## Discussion

An important consideration is the development of insect resistance to the agents employed for their control. To date, there are no reports of the development of resistance to insect pathogenic fungi used in biological control efforts in the field, although it is well known that some insects (even between closely related species) display higher intrinsic resistance to fungal infection than others^45^. Greenhouse and field trials using *B. bassiana* to target the emerald ash borer, *Agrilus planipennis* indicated sublethal effects that included decreased longevity and egg laying, and increased larval development periods^55^. However, in several instances although mortality by these fungal agents can occur, little to no sublethal effects have been observed^56^, or else field conditions have been shown to have the potential to mask or eliminate any significant sublethal effects^57^.

A number of reports have examined the sublethal effects of entomopathogenic fungi on various insects under laboratory conditions. These can impact the insect behavior, i.e. result in reduced feeding^58^,^59^, behavioral fever^60^, or can affect developmental programs including molting and egg laying^24^,^61^,^62^. Exposure to entomopathogenic fungi can sometimes impact the subsequent generation, i.e. progeny that survive fungal exposure. Reduced reproductive fitness of the progeny of *Frankliniella occidentalis* exposed to *B. bassiana* has been noted^63^, and *Aedes aegypti* larvae surviving infection by the narrow host range fungal pathogen *Leptolegnia chapmanii* were disrupted in reproductive success, laying fewer eggs^64^. Reports concerning selection for resistance to entomopathogenic fungi have been mixed. *Galleria mellonella* larvae appeared to develop only minimal resistance to *B. bassiana* even after 25 generations of selection^48^. However, although significant sublethal effects were seen in surviving generations of *B. tabaci* exposed to *B. bassiana*, a gradual reduction in mortality rates was seen in 1^st^ to 3^rd^ generations^41^. Here we observed little to no changes in susceptibility of *B. tabaci* to either low or high doses of *I fumosorosea* after up to 5 generations of selection. In addition, similar numbers of eggs were laid by each respective generation, and they remained equally affected by fungal exposure, i.e. a significant decrease in egg laying was noted after *I. fumosorosea* exposure, however, the effect was similar across the various *B. tabaci* generations examined. These data suggest that in our experimental set-up, no significant resistance development was detected in *B. tabaci* to *I. fumosorosea* infection after several generations under fungal selection. These observations may also be linked to the observed lack of resistance development in that although these tissues were affected during fungal infection, no significant differences were seen across the fungal-selected whitefly generations, suggesting no carry-over effects on fitness with respect to reproduction that might lead to the development of resistance.

Although subtle developmental effects on resistance were not examined in this study, fungal infection was shown to reach the fat body and ovaries of insect adult, resulting in loss of vitellogenin and damage to eggs, an effect readily apparent in *in vitro* assays where these tissues provided a readily utilizable nutrient source supporting fungal growth. In *B. tabaci*, the number of ovarioles per adult varies between 8 and 18 depending on the time (days) after eclosion, with an average of 13–15 within 12 days^65^. Examination of dissected ovaries revealed no significant differences in the total number of ovarioles between uninfected and *I. fumosorosea* treated insects, as well as no differences were seen across the *B. tabaci* generations (1^st^ to 5^th^) whether or not they were infected by *I. fumosorosea*.

As cell-free culture supernatants of *I. fumosorosea* have been shown to be insecticidal^54^, we also investigated any effects these extracts may have on dissected *B. tabaci* fat body and ovaries. Our data showed that these cell-free extracts have toxigenic activity on immune and reproductive tissues, suggesting a means by which both lethal and sub-lethal effects can be exerted in biological control. A number of lethal and sub-lethal effects of cell-free culture supernatants derived from entomopathogenic fungi have been noted. Complex outcomes were reported in the application of cell-fee *M. anisopliae* culture supernatant extracts towards the Mediteranean fruit fly, *Ceratitis capitata*^66^,^67^. Aside from direct toxicity (death), female fly fecundity was reduced upon initial exposure, but little to no effects were seen with respect to the egg fertility or mortality of larvae, although pupae were affected. Here we show that the extracts can result in direct damage to tissues in the absence of the presence of the fungal agent itself. Damage to the fat body, ovaries, and degradation of vitellogenin was observed *in vitro*, indicating the presence of toxic compounds in the culture supernatant. Although further characterization of the extract is warranted, a number of potential candidate compounds exist. Dipicolinic acid (2,6-pyridine dicarboxylic acid, DPA) is known to be an inhibitor of the prophenoloxidase activation^38^ and *I. fumosorosea* produces abundant amounts of DPA^68^. A wide range of potential insecticidal toxic metabolites have been reported in *M. anisopliae* and *B. bassiana*^69^,^70^,^71^ however work in *I. fumosorosea* has lagged behind. Our data confirm the potential for *I. fumosorosea* as an agent for *B. tabaci* control as part of Integrated Pest Management practices and indicate that subsequent generations are likely to continue to be susceptible to the fungus.

## Methods

### Fungal strain and insect maintenance

Strain PF01-N10 of *I. fumosorosea* (CCTCC No. M207088) was originally isolated from a *B. tabaci* nymph^27^. For routine use, *I. fumosorosea* was grown on potato dextrose agar (PDA) and conidia were prepared as described^24^. Conidia of *I. fumosorosea* were counted in a Fuchs-Rosenthal hemocytometer using a compound microscope and adjusted to indicate spore suspensions (10^4^–10^7^ conidia/ml) in 0.05% Tween-80 water. Spore viability was examined by spreading 0.2 ml of the 1 × 10^4^ conidia/ml suspension on PDA and counting the number of germinated cells after 24 h of incubation at room temperature. Cells were considered germinated/viable if the germ tube length was as long as the width of the conidia. Conidial viability was assessed for each batch of cells and only batches estimated to be >95% viable were considered used in experiments. *B. taba*ci was originally collected from plant of *Brassica campestris* L. in Guangzhou and then maintained on the same plant in a greenhouse. Identity of the insect was confirmed by PCR-restriction fragment-length polymorphism analysis and *mtCOI* sequencing as described^72^. The identified *mtCOI* sequence was identical to the GenBank sequence accession no. GQ332577. Second instar *B. taba*ci were reared and prepared as described^24^ for use in fungal virulence bioassays. Plants of *B. campestris* L. were grown in plastic pots and incubated in an artificial climate room at 26 ± 2 °C. Sufficient slow release fertilizer (N/P/K = 13:7:15) was added as required to maintain normal plant growth. Intact plants were maintained in greenhouse and used in this experiment at the six to eight leaf stages with 12 to 18 cm tall.

### Preparation of fungal cell-free culture supernatant ethyl acetate extracts

Fungal conidia (10 ml, 1 × 10^7^ conidia ml^−1^) of *I. fumosorosea* were inoculated into shake cultures in a 1 L flask containing 300 ml of Czapek-Dox broth supplemented with 1% peptone (CZP) and incubated with aeration (180 rpm) at 26 ± 1 °C for 3 d for the production of seed inoculum. The seed inoculum was added to fresh CZP at a 1:9 ratio (v/v, 3 L total volume) and the mixture was incubated with aeration (200 rpm) at 26 ± 1 °C for an additional 6 d after which fungal cells were removed by centrifugation (12000 × g, 15 min) and the cell-free culture supernatant was stored at 4 °C. Metabolites were extracted from the cell-free culture supernatant using ethyl acetate (EthOAc). The cell-free supernatant was mixed with an equal volume of ethyl acetate (1:1, 6 L total final volume) and mixed vigorously for 30 min. The organic phase was collected and concentrated by rotary evaporation (RE −52A, Shanghai Ya Rong Biochemical Instrument Factory, Shanghai, China) under reduced pressure and then stored at −20 °C for use.

### Insect bioassays

*Isaria fumosorosea* insect bioassays were performed using standard methods as described^24^. Newly molted 2^nd^ instars of *B. tabaci* were treated by dipping infested leaves (not excised leaves) into indicated concentrations of *I. fumosorosea* (0, 10^4^ and 10^7^ conidia/ml) for 10 seconds. Each treatment (each concentration) involved at least four leaves with >50 whitefly nymphs per leaf and the entire experiment repeated three times with new batches of insects and new conidial suspensions. Within experiment treatments were performed at the same time, using randomized groups of insects from a single batch. Plants with treated insects/leaves were placed in an air-conditioned room at 26 ± 2 °C, R.H.>85%, L:D = 14:10 h. Treatments were monitored daily until death or new adult emergence, with *B. tabaci* mortality recorded every 24 h after treatment. Dead insects were removed immediately upon detection and placed separately in a clear Petri dish to allow for fungal sporulation on the cadavers. If the sporulation of *I. fumosorosea* was observed, the insect was considered to have been killed as a result of infection by *I. fumosorosea.*

### Selection of *B. tabaci* under *I. fumosorosea* pressure

Newly emerged individual adults (zero generation) were maintained for 1 d and allowed to mate. Mated adults were allowed to lay eggs on *B. campestris* plant leaves (not excised leaves). Eggs were allowed to hatch and the nymph was considered as the 1^st^ generation after selective pressure (only second instar nymph treated with 10^4^ and 10^7^ conidia/ml of *I. fumosorosea*). Insects from the 1^st^ generation were used in experiments re-treated as above to yield the second generation, and the experiment was continued for 5 generations. The nymph mortality of the F1, F3 and F5 generations to various concentrations of *I. fumosorosea* conidial suspensions was examined.

### Measurement effect of *I. fumosorosea* on *B. tabaci* ovipositioning

*B. tabaci* newly emergence adults, derived from nymphs topically treated with indicated concentrations (0, 1 × 10^4^, 1 × 10^7^ conidia/ml) of *I. fumosorosea* conidia as described in the insect bioassays section, were placed in cages (60 × 60 × 60 cm) and allowed to mate for ~1 day. Mated *B. tabaci* pairs were placed separately in a plastic Petri dishes (Ø 9 cm diameter), containing a leaf disk (8 cm diameter) of *B. campestris* L. placed on sterile 2% water agar. Insects (cages and Petri dishes) were kept in an air-conditioned room at 26 ± 2 °C, R.H. >85%, L:D = 14:10 h. Egg production was recorded at 2 d intervals till death of the adult. Fresh leaf discs and bee honeydew were provided every 2 d to maximize ovipositioning and as a food supplement. Each treatment was repeated three times, for each repetition there were total 12–20 pairs of adult (selected at random from the mating adult pairs emergence at different day).

### Microscopic visualization of the *B. tabaci* reproductive system

Newly emergence *B. tabaci* adults, derived from nymph treated as described above, i.e. topically with 0, 1 × 10^4^, 1 × 10^7^ conidia/ml *I. fumosorosea*, were placed in cages (60 × 60 × 60 cm), and allowed to mate. The cages were kept in an air-conditioned room at 26 ± 2 °C, R.H. >85%, L : D = 14 : 10 h. Mated insects were removed at 2, 5 and 8 d post-treatments and their ovaries dissected in PBS buffer solution. The number of ovarioles were observed under a microscope and recorded. Ovary morphology and vitellogenin were observed microscopically. Isolated ovaries were then washed 3 times with sterilize distilled water (ddH_2_O) and placed on Petri dishes containing PDA in order to observe any fungal outgrowth from infected tissues. Each treatment was repeated three times, and 10–15 ovaries were examined per replicate.

In separate experiments, the fat body and ovaries were dissected from un-infected *B. tabaci* adults and placed in a small Petri dish (Ø 3 cm) containing 15 ml of 5 × 10^4^ conidia/ml of freshly harvested *I. fumorososea* conidia in sterilize 0.05% Tween-80 water. Ovaries placed in 15 ml sterilize 0.05% Tween-80 water were used as one control, and conidia (5.0 × 10^4^ conidia/ml in sterile 0.05% Tween-80) placed in a Petri dish were used as another control. The morphology of the fat bodies, ovaries, and associated vitellogenin were observed every 6 h with a microscope. To observe growing hyphae and mycelia, select samples were stained with cotton blue dye (cotton blue 0.05 g, lactic acid 20 ml, phenol 20 g, glycerol 40 ml, ddH_2_O water 20 ml). Each treatment was repeated three times, for each repetition there were total 20 ovaries of adult selected at random from the different treatments to observe.

Fat bodies, ovaries, and associated vitellogenin dissected from un-infected *B. tabaci* were also treated with *I. fumosorosea* ethylacetate fractions (as isolated above). The evaporated *I. fumosorosea* EthOAc fraction was re-suspended in acetone at a concentration of 200 mg/mL and subsequently diluted to a working solution of 10 mg/mL sterilize 0.05% Tween-80. Dissected tissues (ovary), washed with ddH_2_O were placed in a small Petri dish (Ø 3 cm) containing 15 ml of the EthOAc extract working solution. Control samples included isolated tissues placed in 0.05% Tween-80:5% acetone. The morphology of the fat bodies, ovaries, and vitellogenin were observed every 6 h with a microscope.

### Data analyses

Mortality and ovipositing data were analyzed by using one-way analysis of variance (ANOVA). Mean values were compared by Turkey’s student range test (Tukey’s HSD, a = 0.05)^73^.

## Additional Information

**How to cite this article**: Gao, T. *et al*. Lack of resistance development in *Bemisia tabaci* to *Isaria fumosorosea* after multiple generations of selection. *Sci. Rep.* **7**, 42727; doi: 10.1038/srep42727 (2017).

**Publisher's note:** Springer Nature remains neutral with regard to jurisdictional claims in published maps and institutional affiliations.

## Figures and Tables

**Table 1:** Analysis of mortality difference among different generations of *B. tabaci* nymph against *I. fumosorosea.*

*B. tabaci*	4d			8d			14d		
	0	4	7	0	4	7	0	4	7
F1	6.4 ± 1.1 a	22.8 ± 1.9 a	48.8 ± 1.8 a	15.6 ± 0.5 a	43.8 ± 1.2 a	74.3 ± 0.3 a	21.1 ± 1.2 a	50.5 ± 0.9 a	82.1 ± 0.9 a
F3	8.4 ± 0.7 a	19.8 ± 1.6 a	40.5 ± 2.0 a	17.6 ± 0.6 a	40.9 ± 1.0 a	73.8 ± 0.5 a	22.1 ± 1.1 a	49.1 ± 1.7 a	80.1 ± 0.8 a
F5	5.4 ± 1.7 a	19.1 ± 4.2 a	43.3 ± 1.9 a	14.7 ± 2.4 a	41.4 ± 1.1 a	72.3 ± 1.1 a	19.7 ± 1.9 a	50.5 ± 1.5 a	78.7 ± 0.9 a
F, df, *P*	1.549, 2, 0.287	1.370, 2, 0.324	4.811. 2, 0.057	0.994, 2, 0.424	2.319, 2, 0.179	2.240, 2, 0.188	0.694, 2, 0.536	0.398, 2, 0.688	4.046, 2, 0.077

Note: Means ± SE in the same column followed by different letters are significant different (Tukey’s, a = 0.05). 0, 4, 7 mean the concentration of fungal conidia with 0, 1 × 10^4^, 1 × 10^7^ conidia/ml, respectively. F1, F3 and F5 mean the No. 1, 3 and 5 generation of *B. tabaci* nymph, respectively.

**Table 2:** Analysis of oviposition difference among different generations of *B. tabaci* adult against *I. fumosorosea.*

*B. tabaci*	6d			12d			18d		
	0	4	7	0	4	7	0	4	7
F1	42.6 ± 6.6 a	28.5 ± 7.3 a	20.2 ± 3.9 a	73.5 ± 6.1 a	45.8 ± 5.4 a	31.1 ± 2.7 a	92.8 ± 3.7 a	50.8 ± 3.8 a	33.4 ± 3.1 a
F3	44.2 ± 9.4 a	26.7 ± 7.5 a	18.3 ± 2.7 a	81.5 ± 7.3 a	47.7 ± 3.9 a	31.1 ± 3.4 a	92.9 ± 5.9 a	53.3 ± 3.1 a	35.6 ± 3.3 a
F5	34.9 ± 3.6 a	23.0 ± 11.3 a	15.0 ± 7.1 a	82.2 ± 5.8 a	46.3 ± 5.8 a	32.8 ± 6.3 a	94.1 ± 1.9 a	55.1 ± 4.4 a	37.6 ± 3.7 a
F, df, *P*	0.507, 2 0.646	0.101, 2, 0.907	0.294, 2 0.764	0.572, 2 0.616	0.040, 2 0.962	0.050, 2 0.952	0.031, 2 0.970	0.319, 2 0.749	0.377, 2 0.715

Note: Means ± SE in the same column followed by different letters are significant different (Turkey’s, a = 0.05). 0, 4, 7 mean the concentration of fungal conidia with 0, 1 × 10^4^, 1 × 10^7^ conidia/ml, respectively. F1, F3 and F5 mean the No. 1, 3 and 5 generation of *B. tabaci* adult, respectively.

**Table 3:** Analysis of ovariole difference among different generations of *B. tabaci* adult against *I. fumosorosea.*

*B. tabaci*	2d			5d			8d		
	0	4	7	0	4	7	0	4	7
F1	12.1 ± 0.3 a	12.5 ± 0.1 a	12.7 ± 1.1 a	13.5 ± 0.1 a	13.3 ± 0.1 a	12.6 ± 0.2 a	13.4 ± 0.6 a	13.3 ± 0.1 a	13.0 ± 0.6 a
F3	12.8 ± 0.4 a	11.1 ± 1.1 a	11.9 ± 0.5 a	12.3 ± 0.1 a	13.0 ± 0.6 a	12.3 ± 0.5 a	14.0 ±0.8 a	12.9 ± 0.7 a	12.5 ± 1.1 a
F5	13.3 ± 0.7 a	12.2 ± 0.4 a	12.4 ± 0.2 a	13.7 ± 1.1 a	13.0 ± 1.0 a	12.6 ± 0.4 a	13.2 ± 1.0 a	11.8 ± 0.6 a	11.5 ± 1.1 a
F, df, *P*	1.437, 2, 0.358	1.181, 2, 0.418	0.327, 2, 0.744	1.398, 2, 0.372	0.066, 2, 0.938	0.049, 2, 0.953	0.260, 2, 0.787	2.105, 2, 0.268	0.629, 2, 0.591

Note: Means ± SE in the same column followed by different letters are significant different (Turkey’s, a = 0.05). 0, 4, 7 mean the concentration of fungal conidia with 0, 1 × 10^4^, 1 × 10^7^ conidia/ml, respectively. F1, F3 and F5 mean the No. 1, 3 and 5 generation of *B. tabaci* adult, respectively.
